# A Comprehensive Pan-Cancer Analysis Identifies CEP55 as a Potential Oncogene and Novel Therapeutic Target

**DOI:** 10.3390/diagnostics13091613

**Published:** 2023-05-02

**Authors:** Mohamed Samir A. Zaki, Muhammad Alaa Eldeen, Waleed K. Abdulsahib, Ayed A. Shati, Youssef A. Alqahtani, Saleh M. Al-Qahtani, Hassan M. Otifi, Ashwag Asiri, Hesham M. Hassan, Hebatallah Emam Mohammed Ahmed, Samy A. Dawood, Amr Negm, Refaat A. Eid

**Affiliations:** 1 Anatomy Department, College of Medicine, King Khalid University, Abha P.O. Box 62529, Saudi Arabia; 2 Cell Biology, Histology & Genetics Division, Biology Department, Faculty of Science, Zagazig University, Zagazig 44519, Egypt; 3 Pharmacology and Toxicology Department, College of Pharmacy, Al Farahidi University, Baghdad 00965, Iraq; 4 Department of Child Health, College of Medicine, King Khalid University, Abha P.O. Box 62529, Saudi Arabia; 5 Pathology Department, College of Medicine, King Khalid University, Abha P.O. Box 62529, Saudi Arabia; 6 Medical Biochemistry and Molecular Biology Department, Faculty of Medicine, Benha University, Banha 13511, Egypt; 7 Department of Chemistry, College of Science, King Faisal University, Al-Ahsa 31982, Saudi Arabia; 8 Chemistry Department, Faculty of Science, Mansoura University, Mansoura 35516, Egypt

**Keywords:** CEP55, pan-cancer, methylation, prognosis, tumor immunotherapy, biomarker, oncogene

## Abstract

Emerging research findings have shown that a centrosomal protein (*CEP55*) is a potential oncogene in numerous human malignancies. Nevertheless, no pan-cancer analysis has been conducted to investigate the various aspects and behavior of this oncogene in different human cancerous tissues. Numerous databases were investigated to conduct a detailed analysis of CEP55. Initially, we evaluated the expression of *CEP55* in several types of cancers and attempted to find the correlation between that and the stage of the examined malignancies. Then, we conducted a survival analysis to determine the relationship between *CEP55* overexpression in malignancies and the patient’s survival. Furthermore, we examined the genetic alteration forms and the methylation status of this oncogene. Additionally, the interference of *CEP55* expression with immune cell infiltration, the response to various chemotherapeutic agents, and the putative molecular mechanism of *CEP55* in tumorigenesis were investigated. The current study found that *CEP55* was upregulated in cancerous tissues versus normal controls where this upregulation was correlated with a poor prognosis in multiple forms of human cancers. Additionally, it influenced the level of different immune cell infiltration and several chemokines levels in the tumor microenvironment in addition to the response to several antitumor drugs. Herein, we provide an in-depth understanding of the oncogenic activities of CEP55, identifying it as a possible predictive marker as well as a specific target for developing anticancer therapies.

## 1. Introduction

Cancer is universally acknowledged as one of the most critical public health issues with a high rate of mortality [1]. Tumorigenesis is a complicated process influenced by cellular origins, tumor location, and genetic mutations, as well as acquired and inherited molecular abnormalities [2]. Although numerous medications and therapies have been developed for treating cancer, patients have consistently been dissatisfied with the existing options because of significant drug-related side effects, drug resistance, expensive healthcare expenses, and missed targets. Consequently, there is still a pressing must for the investigation of more accurate tumor biomarkers and the detection of potential therapeutic targets. The continuous progression in sequencing technologies has aided in the emergence of numerous public cancer databases, such as the Cancer Genome Atlas (TCGA) [3,4]. The availability of these databases puts our hands on a massive amount of information regarding several types of human cancers. This plentiful data required integrated analysis to study the behavior of a list of detected oncogenes, the mechanism of their tumorigenesis, their expression effect on cases survival, and their roles regarding the response to immunological or chemotherapeutic agents [5].

Centrosome proteins are categorized as scaffold proteins that govern mitotic spindle as well as microtubule tissue, making them crucial to the cell cycle [6]. Centrosome protein 55 (CEP55), also known as FLJ10540 and C10orf3, was first identified as a crucial element of abscission, the final phase of cytoplasmic division that regulates the physical separation of two daughter cells [7]. CEP55 resides in the centrosome throughout the entire cell cycle, the mitotic spindle throughout mitosis, and the midbody throughout cytokinesis [8]. Cytokinesis is tightly regulated during cellular division and involves the CEP55-dependent recruitment of multicomponent subunits to the midbody [8]. CEP55 has been discovered as both a cancer-associated antigen as well as a cancer–testis antigen [9], whereas the latter are proteins that are ordinarily expressed in the testes, but their expression becomes more widespread in cancers [10]. Recent research has demonstrated that CEP55 promotes carcinogenesis and regulates the PI3K/AKT signaling pathway [11]. The link between CEP55 overexpression and the formation and progression of several malignant cancers, particularly breast, stomach, or lung malignancies, is supported by considerable recent findings [12]. The knockdown of CEP55 can not only restrict the viability and proliferation of tumor cells but also induce their apoptosis [12].

While it has been proven that CEP55 performs a significant function in the progression of several forms of cancer, there has been a lack of investigations analyzing the collective aspects of CEP55 in a panel of human cancers, and because of that, we performed this comprehensive pan-cancer analysis. Utilizing the TCGA project and publically available databases, the current study started with a differential analysis to confirm the elevated CEP55 expression in tumor versus normal tissue and correlated this expression with the grade, stage, and metastasis of several human cancers. Following that, we correlated CEP55 expression in tumors with patients’ survival and analyzed the possible mutations and alterations in the targeted gene. Lastly, the study investigated the impact of CEP55 expression on the infiltration of immune cells and the response of a growing tumor to numerous chemotherapeutic drugs where the performed enrichment analysis of CEP55 with tumor interacting and correlated proteins put our hands on possible mechanisms of CEP55 tumorigenesis induction.

## 2. Materials and Methods

### 2.1. CEP55 Differential Expression in Cancerous and Normal Tissues

The current study started by employing two major databases to explore the differential expression of *CEP55* between cancerous and normal tissues. These databases were TIMER2 and GEPIA2 [13]. Additionally, the Human Protein Atlas (HPA) was utilized to explore CEP55 levels in patients diagnosed with various types of cancers [14]. Following the assessment of differential expression, we aimed to analyze the potential correlation between *CEP55* and both tumor stage and grade, where the TISIDB repository was examined for that purpose [15]. The last evaluation in the current stage was the assessment of *CEP55*’s potential roles for metastasis, and the current study utilized the TNMplot web tools for that purpose [16].

### 2.2. CEP55 Differential Protein Expression

After assessing CEP55 differential transcriptional levels in cancerous versus normal tissues, we ran the same assessment but on a protein level where the UALCAN tool was utilized for that purpose [17]. The tumors that exhibited significantly elevated levels of CEP55 protein were analyzed for their IHC images under the HPA web server [18] in order to evaluate the staining status of CEP55 in those tumors versus the corresponding normal tissues. The figures were obtained from the human protein atlas (HPA), and the staining intensity was also reported for every sample in HPA where the staining of CEP55 in cancerous tissue was obtained from the “pathology” tab under HPA, and for comparison, we obtained the staining of the same protein in a corresponding normal tissue in the “tissue” tab under HPA database.

### 2.3. Survival Prognosis Analysis

In order to assess CEP55 as a potential prognosis biomarker, we utilized the “survival analysis” module under the GEPIA2.0 database [13]. This module offers “overall” and “disease-free” survival assessments, where both of which were explored for CEP55 in a panel of human cancers.

### 2.4. Gene Alteration Analysis

For the current assessment, the cBioPortal web server [19] was used to explore the genetic alteration profile of *CEP55*. We explored four slots under the cBioPortal webserver to run the current assessment. Firstly, “Cancer Types Summary” was utilized to analyze the different types of *CEP55* genetic alterations in a panel of human tumors. Secondly, we explored the “Plots” tab to study mutation types of *CEP55* under tumor conditions. Thirdly, the “Mutations” tab was used to find the amino acid sites on *CEP55* with reported mutations. Finally, the “Comparison/Survival” slot was utilized to find the potential correlation between *CEP55* mutations and the clinical outcome. 

### 2.5. CEP55 Differential Phosphorylation and Methylation Assessment

Gene methylation is a major cellular controlling mechanism to regulate the expression of that gene [20], while protein phosphorylation of some oncoproteins has been attributed to their activation and stimulation of cancer progression [21]. Regarding the assessment of *CEP55* differential methylation, the SMART web application [22] was employed, while the UALCAN web server [17] was used to analyze the phosphorylation status of CEP55 in cancerous tissues versus normal ones.

### 2.6. Immune Cell Infiltration Analysis

The tumor microenvironment is characterized by the infiltration of different types of immune cells with various functions. Myeloid-derived suppressor cells (MDSCs) and cancer-associated fibroblasts (CAFs) are considered essential immunosuppressive cells in the tumor microenvironment [23,24]. We asked whether the oncogenic roles of CEP55 are correlated to the infiltration of these immunosuppressive cells; thus, the current study employed the TIMER2 database [25] to study the correlation between CEP55 expression and the infiltration of the above-mentioned cells.

### 2.7. Assessment of Potential Correlation between CEP55 and Different Immunoregulators

CD8 T cells have important roles in fighting against growing cancer; where under tumor conditions, these cells become exhausted, and these exhausted CD8 T cells are characterized by the expression of several immune checkpoints [26]. At this stage, we aimed to study the possible correlation between CEP55 upregulation in cancerous tissues and the levels of different immune checkpoints (CTLA4, PD1, LAG3, and TIGIT) where the TISIDB repository was employed for that purpose [15]. Chemokines are immune molecules with different roles in the tumor microenvironment; where some of them were associated with tumor suppressive functions, such as CCL14 [27], others were mentioned for their tumor-stimulating activities, for example, CXCL8 [28]. Due to their important roles in the tumor microenvironment, we explored the potential correlation between CEP55 and both CCL14 and CXCL8 also through the utilization of the TISIDB repository [15].

### 2.8. Association of CEP55 with TMB and MSI

Tumor mutational burden (TMB) and microsatellite instability (MSI) were nominated as predictors of response to immune checkpoint inhibitors (ICI) [29]. Hence, the association between CEP55 and both TMB and MSI is important to predict the response to ICI in patients with elevated levels of CEP55, where we used the Sangerbox platform [30] to run this assessment. 

### 2.9. Interference of CEP55 with the Activity of Chemotherapeutic Drugs

Chemotherapeutic agents represent an important choice for cancer treatment [31]. At the current stage, we utilized CellMiner [32] to explore the possible correlation between CEP55 expression and the activity of several chemotherapeutic agents.

### 2.10. CEP55 Interacting Network

In this final assessment, we aimed to detect and analyze CEP55-interacting and correlated proteins. Regarding interacting ones, the STRING database [33] was utilized for that detection, while the GEPIA2 database was employed to detect the top 100 proteins correlated with CEP55 in the tumor microenvironment. Moreover, the “Gene Corr” tab under the TIMER2 web server was used to generate a heatmap demonstrating and confirming the correlation between CEP55 and the top 5 correlated proteins (as predicted from the GEPIA2 database). Following that, Venn diagram viewer (http://bioinformatics.psb.ugent.be/webtools/Venn/, accessed on 28 January 2023) was used to find the common interacting and correlated proteins with CEP55 where after duplicates removal, the generated protein list was submitted to the DAVID database [34] to perform enrichment analysis.

## 3. Results

A list of cancer names and abbreviations mentioned in the current study is demonstrated in Supplementary Table S1.

### 3.1. Multiple Human Cancers Experienced Upregulation in CEP55 Expression

**Figure 1:**
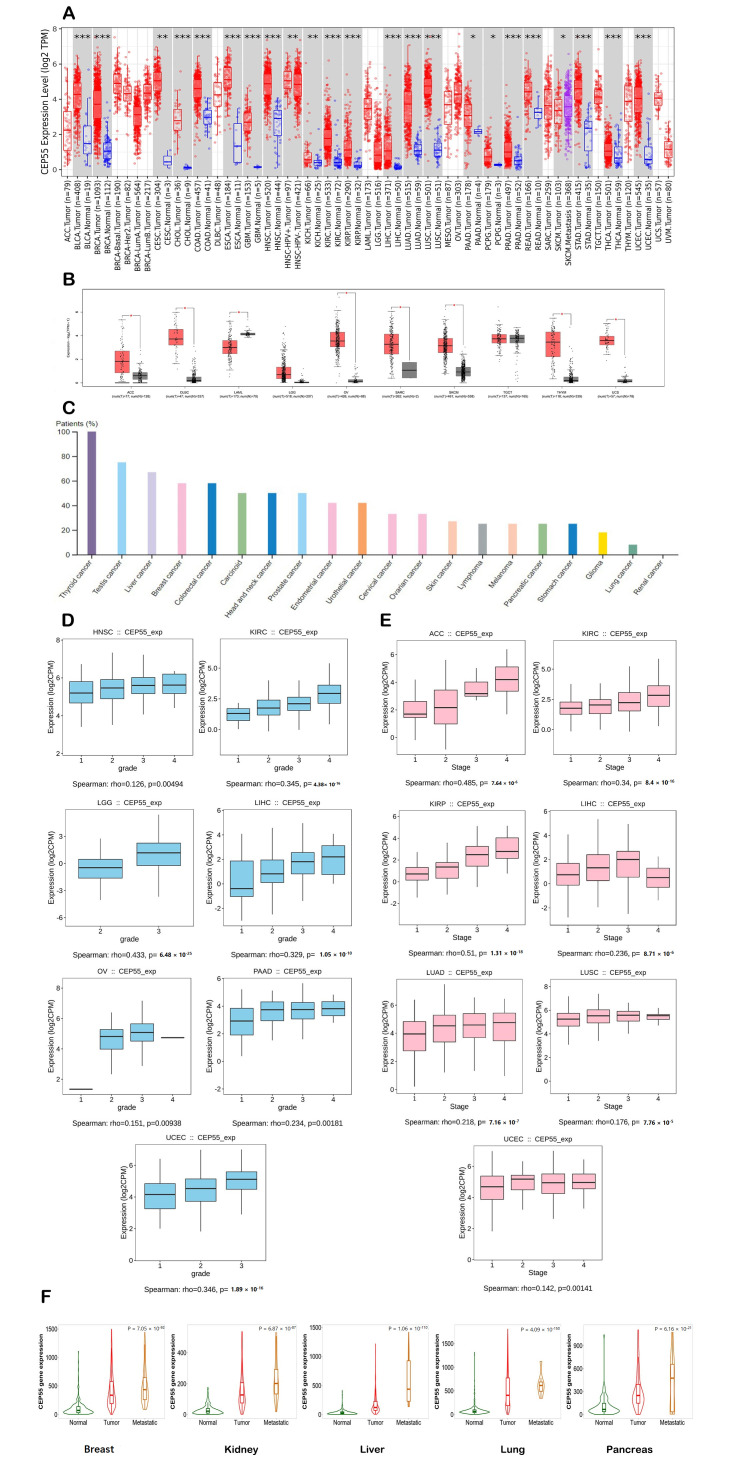
Differential expression of *CEP55* in a panel of human tumors versus normal tissues and the correlation with tumor grade, stage, and metastasis. (**A**) Differential expression of *CEP55* based on the TIMER2.0 output; (**B**) differential expression of *CEP55* based on the GEPIA2 database output (**C**) levels of *CEP55* in several human cancers examined by HPA; (**D**,**E**) tumors experiencing a positive correlation between *CEP55* expression and tumor grade and stage, respectively; (**F**) tumors experiencing a consistent positive relationship between *CEP55* expression and tumor metastasis; *: *p*-value < 0.05; **: *p*-value < 0.01; ***: *p*-value < 0.001.

We first used TIMER2 to assess the differential expression of *CEP55* between cancerous tissues and normal ones in a panel of human tumors. It was found that BLCA, BRCA, CHOL, COAD, ESCA GBM, HNSC, KIRC, KIRP, LIHC, LUAD, LUSC, PRAD, READ, STAD, THCA, UCEC (*p* < 0.001), CESC, KICH (*p* < 0.01), PAAD, and PCPG (*p* < 0.05) experienced significant upregulation in *CEPP55* expression (Figure 1A). Following that, we utilized the GEPIA2 database to analyze the differential expression for the tumors that lack “normal tissue score” for expression comparison in the TIMER2 database. It was found that seven tumors, namely, ACC, DLBC, OV, SARC, SKCM, THYM, and UCS, demonstrated significant upregulation in *CEP55* expression versus the corresponding normal tissues (Figure 1B). It is worth mentioning that only one tumor, namely, LAML, experienced the opposite pattern where *CEP55* expression was significantly higher in normal tissue versus cancerous one. Analyzing cancers by tissue revealed that the CEP55 protein was overexpressed in several human cancers (Figure 1C), where patients with thyroid, testis, and liver cancers came at the top of this list. As a next step, we investigated the correlation between *CEP55* overexpression in tumor tissue and tumor grade and stage. The output from the TISDIB server revealed that KIRC, LGG, LIHC, UCEC (*p* < 0.001), HNSC, OV, and PAAD (*p* < 0.01) experienced a positive correlation between *CEP55* expression and tumor grade (Figure 1D). Moving to the tumor stage, ACC, KIRC, KIRP, LIHC, LUAD, UCEC, and LUSC (*p* < 0.001) showed a positive correlation between CEP55 expression and tumor stage (Figure 1E). Finally, the correlation between *CEP55* expression and metastasis was studied based on the data of the TNMplot server, where the upregulation of *CEP55* expression was positively correlated with the metastasis progression in the tumors of the breast, kidney, liver, lung, and pancreas (Figure 1F).

### 3.2. CEP55 Demonstrated Elevated Protein Levels in Cancerous Tissue

**Figure 2:**
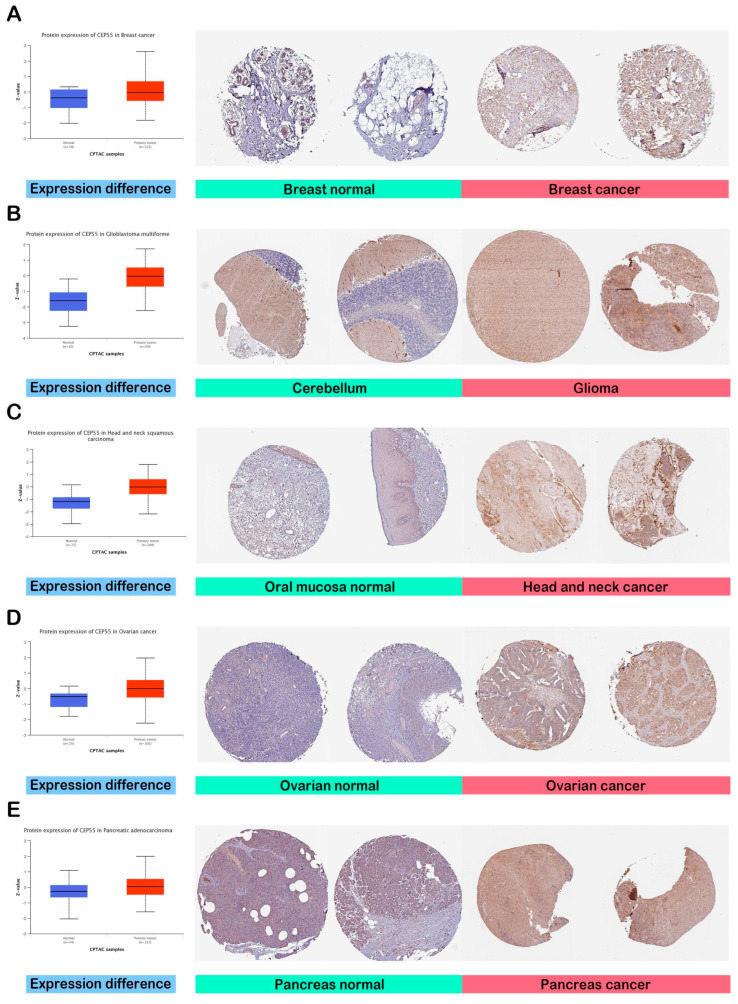
Assessment of CEP55 protein levels in terms of differential protein expression between cancerous and normal tissues in addition to the IHC staining. (**A**) Breast; (**B**) glioblastoma multiforme; (**C**) HNSC; (**D**) ovarian; (**E**) pancreatic cancers.

The previous stage of the analysis revealed increased levels of CEP55 mRNA in cancerous tissues versus normal ones. At this stage, we aimed to analyze the potential elevation of CEP55 at a protein level. Figure 2A–E demonstrate that CEP55 protein expression was significantly elevated in breast cancer, glioblastoma multiforme, HNSC, ovarian cancer, and PAAD in comparison to the corresponding normal tissues. In addition to that, we explored the IHC figures of these tumors and their correlated normal tissues, and the findings matched with the results of differential protein expression as the staining for CEP55 was low or even undetected in normal tissue, while it was moderate to intense in the corresponding assessed tumors (Figure 2A–E).

### 3.3. Interference of CEP55 with the Clinical Outcome

**Figure 3:**
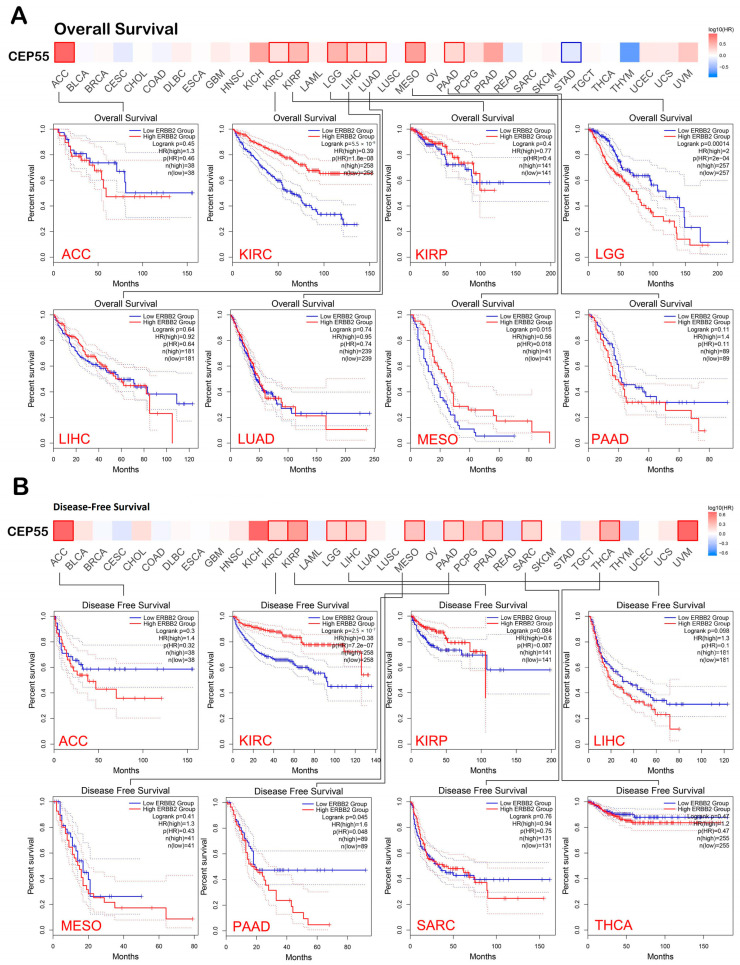
A heat map with detailed survival graphs of the tumors that experienced a positive correlation between CEP55 level and the clinical outcome under the studied modules. (**A**) Overall survival; (**B**) disease-free survival.

At this stage, the GEPIA2 database was employed to study the potential effect of CEP55 on the patients’ survival under two models, namely, “overall survival” and “disease-free survival”. Starting with overall survival, eight tumors, namely, KIRC, KIRP, LUAD, PAAD (*p* < 0.01), ACC, LGG, LIHC, and MESO (*p* < 0.001), exhibited a positive correlation between CEP55 levels and the clinical outcome (Figure 3A). Moving to disease-free survival, 11 tumors, namely, KIRC, MESO, PAAD, SARC (*p* < 0.05), LGG, PRAD, THCA, UVM (*p* < 0.01), ACC, KIRP, and LIHC (*p* < 0.001), demonstrated a positive correlation between the level of CEP55 and the clinical outcome (Figure 3B). Collectively, seven tumors (KIRC, KIRP, PAAD, ACC, LGG, LIHC, and MESO) experienced a positive correlation between CEP55 and the patients’ survival under both of the studied modules, a finding that supports the reliance on CEP55 as a potential prognosis biomarker.

### 3.4. Analysis of Genetic Mutations in CEP55

**Figure 4:**
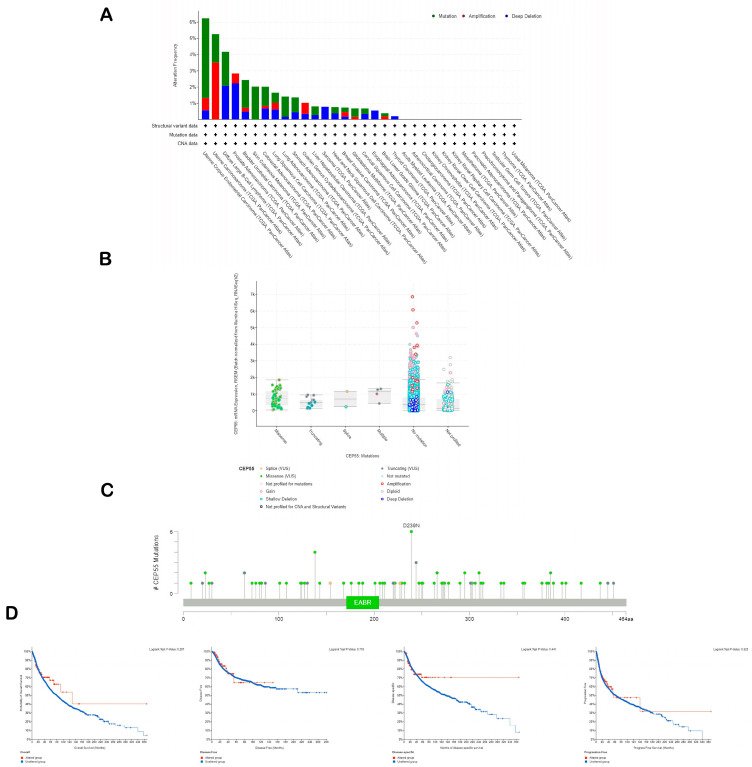
Analysis of CEP55 genetic alterations. (**A**) The frequency of CEP55 reported genetic alterations in a panel of human tumors; (**B**) reported mutation types for CEP55; (**C**) sites of CEP55 with reported genetic alterations; (**D**) survival graphs representing the correlation between CEP55 genetic alterations and the clinical outcome.

The cBioPortal website was utilized to study the genetic alterations in *CEP55*. Firstly, the types of *CEP55* genetic alterations were highly variable between the studied tumors. We cannot simply detect one type of genetic alteration as a dominant one. The mutation was the dominant alteration reported for *CEP55* in UCEC, BLCA, SKCM, and LUAD. On the other hand, amplification was the most reported *CEP55* alteration in UCS and OV, while deep deletion represented the highest percentage of *CEP55* genetic alterations in PRAD, SARC, and ESCA (Figure 4A). Moving to the assessment of the mutation types, missense mutations were found to be the most reported ones, where the amino acid number 239 was the highest reported site in *CEP55* for missense mutation with six reported cases (Figure 4B,C). The final step in the current assessment was the evaluation of *CEP55* alterations’ effect on the clinical outcome where the output from the cBioPortal revealed that there is no correlation between the studied factors in terms of overall, disease-free, disease-specific, and progress-free survivals (Figure 4D). 

### 3.5. Differential Phosphorylation and Methylation Analysis of CEP55 

**Figure 5:**
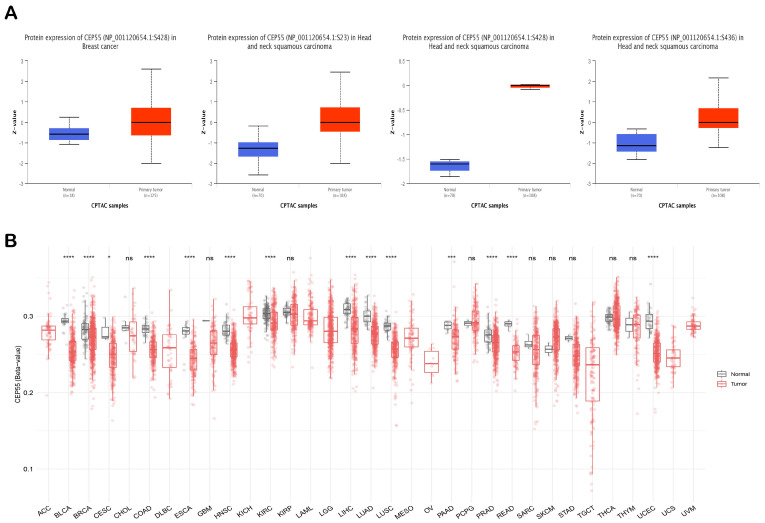
Investigation of the differential phosphorylation and methylation status of CEP55 in malignant vs. normal tissue samples. (**A**) Tumors and sites that experienced significant hyperphosphorylation in tumor tissue versus normal one; (**B**) CEP55 CpG-aggregated methylation status in normal tissues versus cancerous ones; *: *p*-value < 0.05; ***: *p*-value < 0.01; ****: *p*-value < 0.001.

Phosphorylation and methylation status have been correlated with protein activity and gene regulation, respectively. Regarding phosphorylation, one site (S428) on the CEP55 amino acid sequence was found to be significantly phosphorylated in HNSC and breast cancer. Moreover, another two sites on CEP55, namely, S23 and S436, were also significantly phosphorylated in HNSC versus corresponding normal tissue (Figure 5A). Moving to the methylation analysis, *CEP55* CpG-aggregated was found to be significantly hypomethylated in BLCA, BRCA, CESC, COAD, ESCA, HNSC, KIRC, LIHC, LUAD, LUSC, PAAD, PRAD, READ, and UCEC (Figure 5B). Collectively, phosphorylation and methylation can be considered potential cellular mechanisms that control the activity and expression of CEP55 in normal tissue versus cancerous ones. 

### 3.6. CEP55 Correlates with Immune Infiltration in Several Tumor Types

**Figure 6:**
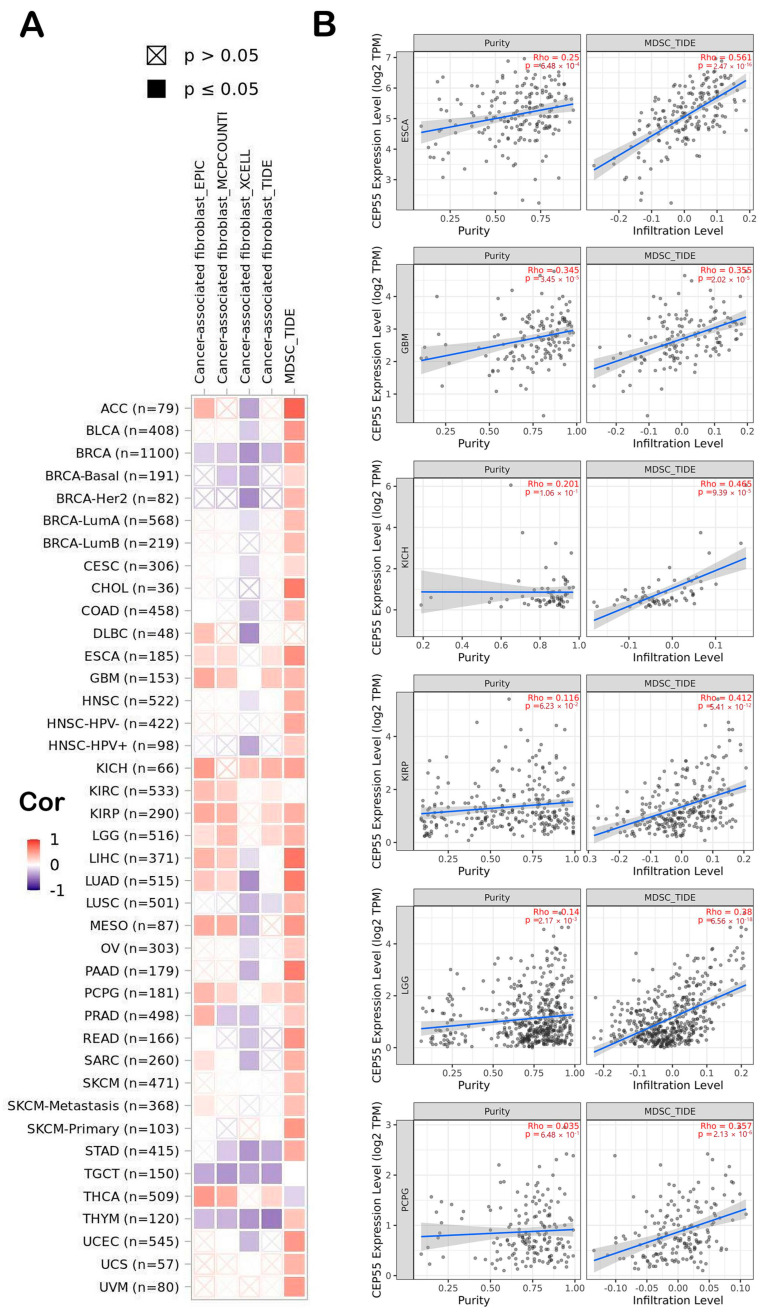
(**A**) A heatmap demonstrating the correlation between CEP55 and the infiltration of 2 immunosuppressive cells (MDSCs and CAFs); (**B**) scatter plots of the tumors that experienced a positive correlation between CEP55 and infiltration of both MDSCs and CAFs. The plots show the correlation with MDSC infiltration.

Immunological characteristics of the cells that infiltrate the tumor can largely affect the clinical outcome. From the previous sections, we found that CEP55 is highly expressed in tumor tissue and predicts a poor clinical outcome; therefore, we aimed to correlate that with the immune cells that preferentially infiltrate the tumor in a correlation with CEP55 upregulation where we targeted two types of cells with reported immunosuppressive activity, namely, MDSCs and CAFs. Starting with MDSCs, around 90% of the analyzed tumors demonstrated a positive correlation between MDSC and CEP55, with only one tumor, THCA, that showed a negative correlation between the same factors (Figure 6A). Moving to CAFs, eight tumors, namely, ESCA, GBM, KICH, KIRC, KIRP, LGG, PCPG, and THCA, showed a positive correlation between CEP55 and CAFs infiltration. Collectively, six tumors (ESCA, GBM, KICH, KIRP, LGG, and PCPG) showed a positive correlation between CEP55 and the infiltration of both MDSCs and CAFs, where Figure 6B illustrates the scatter plots that show the correlation between CEP55 and MDSCs infiltration in these six tumors.

### 3.7. CEP55 Positively Correlates with Immune Checkpoints and Immunosuppressive Chemokines

**Figure 7:**
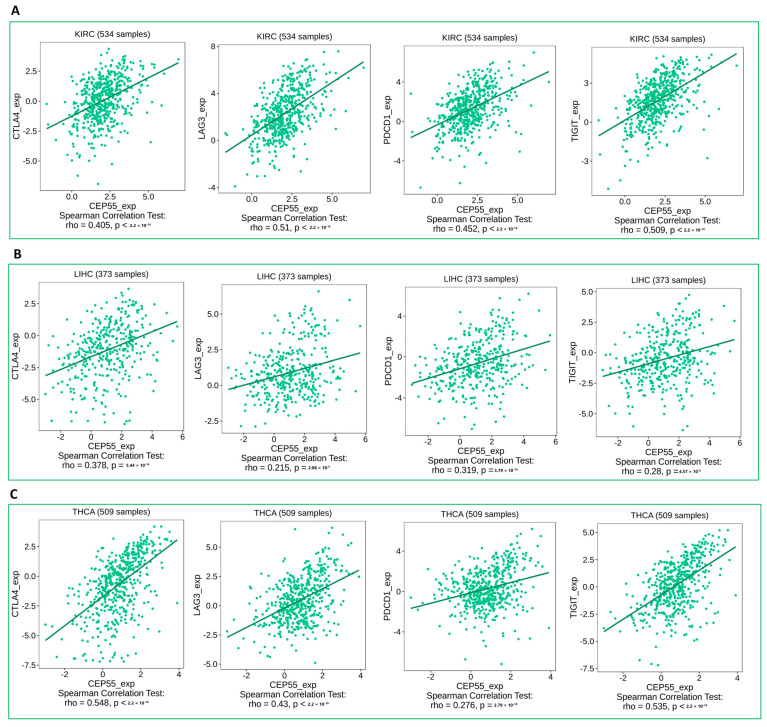
Plots showing the positive correlation between CEP55 expression and the levels of immune checkpoint inhibitors (CTLA4, LAG3, PD1, and TIGIT) in (**A**) KIRC, (**B**) LIHC, (**C**) THCA.

**Figure 8:**
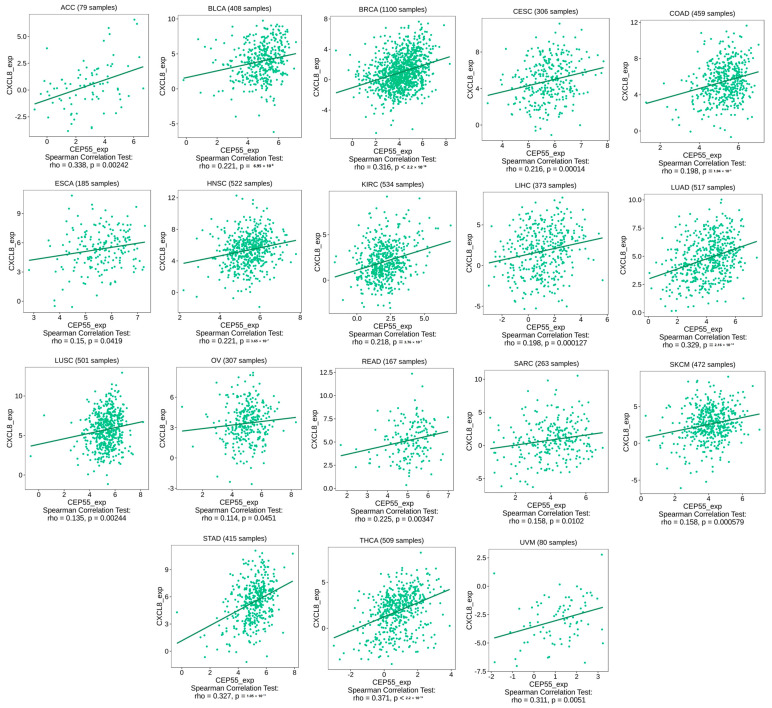
Tumors that experienced a positive correlation between the expression levels of CEP55 and CXCL8.

Under the chronic condition of stimulation in cancer, CD8 T cells start to express immune checkpoints that limit the cytotoxic ability of that cell. Moreover, several chemokines have been reported to have immunosuppressive activity under tumor conditions, where CXCL8 is an example of these chemokines. Starting with immune checkpoints, four of them, CTLA4, LAG3, PD-1, and TIGIT (that have been targeted as antitumor therapeutic targets), have shown a positive correlation with CEP55 expression in three different tumors, namely, KIRC, LIHC, and THCA (Figure 7A–C). This finding defines the exhaustion induction roles of CEP55 on CD8 T cells as a possible mechanism for tumor progression under elevated levels of CEP55 in cancerous tissues. Moving to the chemokine correlations, CEP55 has shown a positive correlation with the immunosuppressive chemokine CXCL8 in approximately 60% of the assessed tumors (Figure 8). Matching these results, CEP55 exhibited a negative correlation with CCL14, a chemokine that exhibited antitumor activity, in around 85% of the analyzed tumors. 

### 3.8. CEP55 Expression Patterns Correlate with TMB and MSI

**Figure 9:**
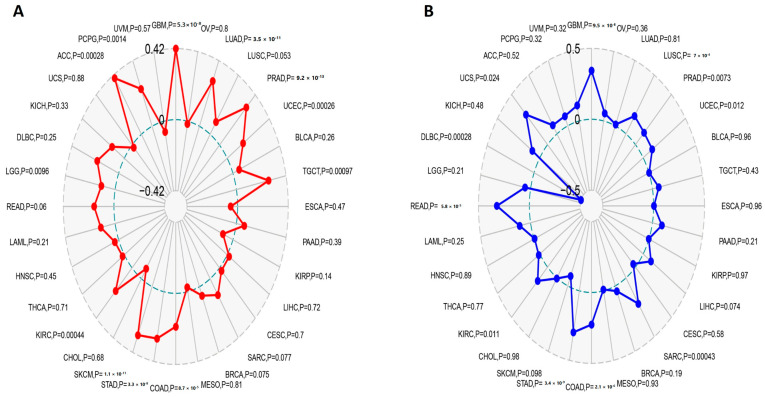
Correlations of CEP55 expression with immune checkpoints, MSI, and TMB. (**A**,**B**) Radar charts illustrating the overlaps of CEP55 with TMB and MSI, respectively.

TMB and MSI have been nominated as genomic biomarkers for cases that have the potential to be responsive to immune checkpoint inhibitors. Hence, it was important to analyze the correlation between CEP55 and TMB, and MSI. Firstly, LGG, PCPG (*p* < 0.01), GBM, LUAD, PRAD, UCEC, TGCT, COAD, STAD, SKCM, KIRP, and ACC (*p* < 0.001) showed a positive correlation between CEP55 and TMB (Figure 9A). Additionally, UCEC, KIRC, UCS (*p* < 0.05) PRAD (*p* < 0.01), GBM, LUSC, SARC, COAD, STAD, and READ (*p* < 0.001) exhibited a positive correlation between CEP55 and MSI (Figure 9B). Collectively, six tumors, namely, GBM, PRAD, UCEC, COAD, STAD, and KIRC, demonstrated a positive correlation between CEP55 and both TMB and MSI; thus, CEP55 can be used as a biomarker for the response of the patients of these tumors to immune checkpoint inhibitors.

### 3.9. CEP55 Negatively Correlated with IC50 of Several Chemotherapeutic Drugs

**Figure 10:**
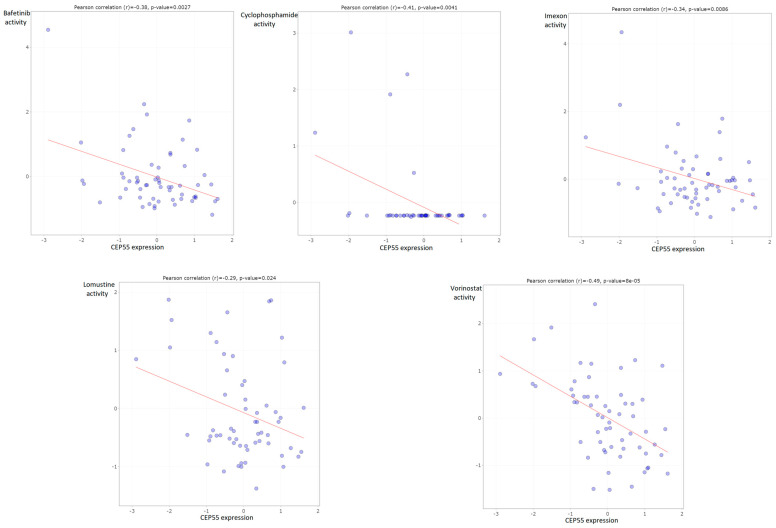
Plots of chemotherapeutic agents showed a negative correlation between activity and CEP55 expression.

After analyzing the correlation between CEP55 expression and different immunological components, we asked if CEP55 levels could interfere with the activity of chemotherapeutic antitumor agents to obtain guidance for chemotherapeutic selection for cancer patients with high CEP levels. Our analysis revealed that CEP55 expression was negatively correlated with the IC50 of five drugs, namely, bafetinib, cyclophosphamide, imexon, lomustine, and vorinostat (Figure 10).

### 3.10. Enrichment Analysis for CEP55 Interacted-Correlated Proteins

**Figure 11:**
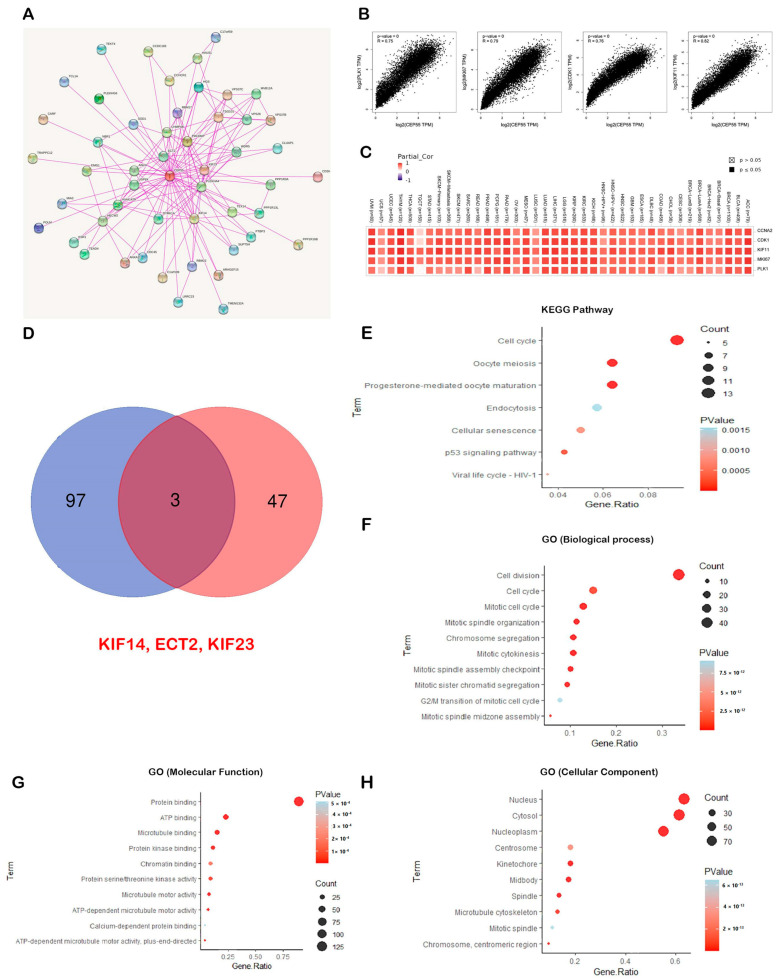
CEP55-proteins network and their enrichment analysis. (**A**) Map representing top 50 CEP55-interacting proteins; (**B**) plots demonstrating the positive correlation between CEP55 and the top 5 correlated proteins; (**C**) heatmap demonstrating the positive correlation between CEP55 and the top 5 correlated proteins in a panel of human cancers; (**D**) Venn diagram demonstrating the common CEP55-interacting and correlated proteins; (**E**–**H**) KEGG/GO enrichment analysis for the combined list of CEP55-interacting and correlated proteins.

According to the above-mentioned findings, CEP55 directly correlates with cancer patient survival and interferes with several immunological components; hence, the potential molecular mechanism of CEP55 oncogenic functions should be studied. Here, we started by exploring the top 50 interacting proteins with CEP55 relying on the data from the STRING database (Figure 11A). Following that, the top 100 correlated proteins with CEP55 were collected from the GEPIA2 database, where the proteins KIF11, MK167, CDK1, PLK1, and CCNA2 represented the top 5 proteins correlated with CEP55 in the tumor microenvironment that influence immune cells in TME (Figure 11B). For more confirmation, the “Gene Corr” module in the TIMER2 webserver was employed to investigate the correlation between CEP55 and those top five proteins in a panel of human tumors, where a generated heatmap (Figure 11C) confirmed the significant positive correlation in all of the analyzed tumors (except for CEP55 and PLK1 in TGCT, the correlation was insignificant). Using a Venn diagram, we determined that KIF14, ECT2, and KIF23 were the common members of the intersection of the CEP55 interacting and correlated proteins (Figure 11D). Enrichment analysis for the combined two lists (CEP55-interacting and CEP55-correlated proteins) after duplicate removal was the last stage of analysis in the current study. Regarding KEGG pathway analysis, “cell cycle” was generated as the top pathway for the analyzed genes, which could explain the mechanism of CEP55 roles in carcinogenesis as it interacts with specific proteins to stimulate the cell cycle (Figure 11E). Furthermore, GO enrichment analysis indicates that CEP55-interacting and correlated proteins are primarily associated with the biological mechanisms of “cell division”, “cell cycle”, and “mitotic cell cycle” (Figure 11F); with the molecular functions of “protein binding”, “ATP binding”, and “microtubule binding” (Figure 11G); and with the cellular components of the “nucleus”, “cytosol”, and “nucleoplasm” (Figure 11H).

## 4. Discussion

Cancer is categorized as a major cause of human death, where interventions such as routine surgical resection, radiation, immunotherapy, and chemotherapy have been utilized, but several individuals have failed to receive a positive response and are still suffering [35]. CEP55 takes part in cytokinesis, and its abnormal expression is associated with genomic instability, a hallmark of cancer [36]. Several studies have analyzed the oncogenic roles of CEP55 in individual tumors. CEP55 upregulation was correlated with the improved phosphorylation of AKT, which finally promoted cell proliferation in gastric carcinoma [37]. CEP55 can also activate the PI3K/AKT pathway as an oncogenic mechanism in hepatocellular carcinoma [38], and through the same mechanism, CEP55 was found to activate the proliferation of human glioma U251 cells [39], osteosarcoma [40], and esophageal squamous cell carcinoma [41]. The activation of cell motility via JAK2–STAT3–MMPs cascade was another reported mechanism for the oncogenic activity of CEP55 in hepatocellular carcinoma [42]. CEP55 was also negatively correlated with the clinical outcome in multiple human tumors, including liver cancer [43], non-small cell lung cancer [44], esophageal squamous cell carcinoma [45], and ovarian epithelial carcinoma [46]. It was not a surprise that the knockdown of CEP55 can suppress cell proliferation [47], and modulating its overexpression through microRNA targeting inhibited cell proliferation and migration [48]. As mentioned above, several studies have investigated the oncogenic roles and effects of CEP55 in individual human tumors; therefore, we directed the current study to analyze the complex behavior of CEP55 in a pan-cancer model.

In the current study, CEP55 was found to be upregulated in most of the analyzed human cancers versus the corresponding normal tissues, which is considered a traditional feature of oncogenic proteins [49]. Cancer stage and grade are essential values to predict the clinical behavior of malignancies and select the most appropriate therapies [50]. Moreover, cancer metastasis is a hallmark of cancer that was considered the key cause of failure of cancer therapy and mortality [51]. As a result, it was important, after confirming the upregulation status of CEP55 in cancerous tissues, to ask for the consequences of this upregulation on tumor stage, grade, and metastasis. The current study reported that KIRC, LGG, LIHC, UCEC, HNSC, OV, and PAAD showed a positive correlation between CEP55 expression and tumor grade, while ACC, KIRC, KIRP, LIHC, LUAD, UCEC, and LUSC showed the same correlation between CEP55 expression and tumor stage. Finally, the upregulation in CEP55 expression was positively correlated with the metastasis progression in tumors of the breast, kidney, liver, lung, and pancreas. Another concern of the current study was the effect of CEP55 on the clinical outcome. Survival analysis is an essential investigation point in cancer studies, as it reflects the state of disease progression and the individual’s response to medical interventions [52]. The output from the GEPIA2 database revealed that seven tumors, namely, KIRC, KIRP, PAAD, ACC, LGG, LIHC, and MESO, experienced a positive correlation between CEP55 and the patients’ survival in two studied models (overall and disease-free survivals). This result matches with the recently reported role of CEP55 overexpression in the poor prognosis of cancer patients [53], a point that nominates CEP55 as a potential prognostic biomarker in multiple human tumors.

The human cell is able to control the expression status of its genes through several mechanisms, where gene methylation represents a major mechanism for that regulation [54]. As a general rule, tumor suppressor genes are hypermethylated to be silenced under tumor conditions [55]. On the other hand, genes with oncogenic rules usually experience a hypomethylated status to be activated in the state of cancer progression [56]. Therefore, gene methylation assessment is an important research point in cancer studies where it has been correlated with the early detection of cancer [57]. A hypomethylation status has been reported for several oncogenes, including AQP1 in salivary gland carcinoma [58], LINE-1 in colorectal cancer [59], and ELMO3 in lung cancer [60]. Matching with these oncogenes, CEP55 was found to be significantly hypomethylated in BLCA, BRCA, CESC, COAD, ESCA, HNSC, KIRC, LIHC, LUAD, LUSC, PAAD, PRAD, READ, and UCEC versus normal tissues. Protein phosphorylation is a major cellular post-translational modification that represents a crucial regulatory mechanism for many proteins and receptors [61]. Malfunction of specific proteins’ phosphorylation has been detected in several tumors [62]. Due to its significance, phosphoproteomic analysis has been employed to detect certain therapeutic targets for antitumor treatments [63]. In the current study, one site (S428) on the CEP55 amino acid sequence was found to be significantly phosphorylated in HNSC and breast cancer, and another two sites, namely, S23 and S436, were also significantly phosphorylated in HNSC versus corresponding normal tissue, a feature that can be connected with the hyperactivity of CEP55 that finally led to tumor progression.

The tumor microenvironment is characterized by the infiltration of lymphocytes with varying functions. The interference of oncoproteins with the amount, kind, and phenotype of the infiltrating immune cells could be a possible mechanism for cancer progression, where some oncoproteins are correlated with the enhanced infiltration of components with immunosuppressive roles [64]. MDSCs and CAFs are two major cells that infiltrate the tumor and have immunosuppressive activity [65]. MDSCs have different variable roles starting from the inhibition of antitumor immune reactions and stimulating cancer growth and metastasis [66] and moving to the promotion of resistance against antitumor drugs [67]. CAFs are highly enriched cells in the tumor microenvironment, functioning to stimulate tumor progression [68], where a deep understanding of CAFs biology can be employed to find out novel antitumor targets [69]. Due to their immunosuppressive roles, we expected that CEP55 could enhance the infiltration of these cells as a possible mechanism for its tumorigenesis functions. Matching with that, our analysis revealed six tumors, namely, ESCA, GBM, KICH, KIRP, LGG, and PCPG, with a positive correlation between CEP55 and the infiltration of both MDSCs and CAFs. CD8 T cell was another cell that was correlated with CEP55 in the current study, as we aimed to find the possible correlation between CEP55 and the CD8 phenotype (not the infiltration like MDSCs and CAFs). CD8 T cells have major cytotoxic functions, but under chronic stimulation conditions, such as cancer, these cells become exhausted [70]. A hallmark of CD8 exhaustion is the expression of surface molecules called immune checkpoints that act as a brake to hinder the cytotoxic ability of activated CD8 [71]. These checkpoints have been targeted by monoclonal antibodies, an approach that has revolutionized the branch of tumor immunotherapy [72]. The current study asked if CEP55 can contribute to the exhaustion of CD8 T cells where three tumors, namely, KIRC, LIHC, and THCA, exhibited a positive correlation between CEP55 level and the expression of the checkpoints PD1, CTLA4, LAG3, and TIGIT, which are important indicators of CD8 T cell exhaustion. After investigating the roles of CEP55 in the infiltration of immunosuppressive cells and the hindrance of cytotoxic cell activity, the current study asked if CEP55 has extended roles that could include other immune components, such as chemokines. CXCL8 is a chemokine that has tumor-stimulating functions. It is released from tumor-associated macrophages and improves the metastatic potential of a tumor [73]. It was also correlated with the progression and invasion of esophageal squamous cell carcinoma [74]. On the other hand, CCL14 is a chemokine with antitumor activities. It modulates the cell cycle and promotes apoptosis [75]. Moreover, the upregulation of CCL14 was associated with a more favorable prognosis in ovarian cancer patients [76]. Matching with its oncogenic roles, CEP55 was found to be positively correlated with CXCL8 and negatively correlated with CCL14 in most of the current study-analyzed tumors. These results put our hand on a valuable component of the immune system, besides the cells, that can be modified by the action of CEP55 as a part of its oncogenic roles.

The final assessment performed in the current study was the enrichment analysis for CEP55-interacting and correlated genes. The combined gene list was enriched for cell cycle and cell division for molecular function enrichment, which confirms the oncogenic behavior of CEP55 by stimulating cell division in cancerous tissues. It is worth mentioning that three proteins, namely, KIF14, KIF23, and ECT2, were found to be common in the two lists of CEP55-interacting and CEP55-correlated proteins. KIF14 promotes tumor progression and metastasis and acts as a predictor of poor prognosis in human gastric cancer [77]. It also stimulates AKT phosphorylation and contributes to chemoresistance in breast cancer [78]. In addition to that, KIF14 promotes tumor invasiveness and correlates with poor prognosis in prostate cancer [79]. Moving to KIF23, it also stimulates cell proliferation and is associated with cancer progression [80,81,82]. The oncogenic behavior of these shared proteins was extended to ECT2; ECT2 regulates the Rho/ERK signaling axis to promote tumor recurrence [83] and is associated with poor clinical outcomes [84]. This interacting network of CEP55 should be deeply investigated in future studies, as it could represent a potential pathway for antitumor therapeutic targeting.

## 5. Conclusions

CEP55 is an oncoprotein that demonstrated upregulation in cancerous tissues in multiple human tumors, and this upregulation was positively correlated with tumor stage, grade, metastasis, and poor clinical outcomes. Here, we investigated the interference of CEP55 with multiple cells and immune components, where our findings suggest this interference as a potential mechanism for the CEP55 oncogenic acts. We also investigated the pathways for CEP55 molecular interactions and nominated its interaction with three proteins—KIF14, KIF23, and ECT2—as a potential pathway for tumor progression that can be targeted for therapeutic interventions where computational tools can also be employed through a cheminformatics approach to analyze the binding of several molecules to these targets, an approach that could be translated later to effective anticancer drugs.

## Data Availability

The data presented in this study are available on request from the corresponding author.

## Supplementary Materials

The following supporting information can be downloaded at: https://www.mdpi.com/article/10.3390/diagnostics13091613/s1, Table S1: The abbreviations and the full name of analyzed tumors in the current study.

## References

[1] Torre L.A., Siegel R.L., Ward E.M., Jemal A. (2016). Global Cancer Incidence and Mortality Rates and Trends—An Update. Cancer Epidemiol. Biomark. Prev..

[2] Tabassum D.P., Polyak K. (2015). Tumorigenesis: It takes a village. Nat. Rev. Cancer.

[3] Hutter C., Zenklusen J.C. (2018). The Cancer Genome Atlas: Creating Lasting Value beyond Its Data. Cell.

[4] Edgar R., Domrachev M., Lash A.E. (2002). Gene Expression Omnibus: NCBI gene expression and hybridization array data repository. Nucleic Acids Res..

[5] Cui K., Wu X., Gong L., Yao S., Sun S., Liu B., Zhou M., Yin Y., Huang Z. (2021). Comprehensive Characterization of Integrin Subunit Genes in Human Cancers. Front. Oncol..

[6] Chavali P.L., Pütz M., Gergely F. (2014). Small organelle, big responsibility: The role of centrosomes in development and disease. Philos. Trans. R. Soc. B Biol. Sci..

[7] Chang Y.-C., Wu C.-H., Yen T.-C., Ouyang P. (2012). Centrosomal Protein 55 (Cep55) Stability Is Negatively Regulated by p53 Protein through Polo-like Kinase 1 (Plk1). J. Biol. Chem..

[8] Jeffery J., Sinha D., Srihari S., Kalimutho M., Khanna K.K. (2016). Beyond cytokinesis: The emerging roles of CEP55 in tumorigenesis. Oncogene.

[9] Tandon D., Banerjee M. (2020). Centrosomal protein 55: A new paradigm in tumorigenesis. Eur. J. Cell Biol..

[10] Shiraishi T., Getzenberg R.H., Kulkarni P. (2012). Cancer/testis antigens: Novel tools for discerning aggressive and non-aggressive prostate cancer. Asian J. Androl..

[11] Zhu H., Chen D., Tang J., Huang C., Lv S., Wang D., Li G. (2018). Overexpression of centrosomal protein 55 regulates the proliferation of glioma cell and mediates proliferation promoted by EGFRvIII in glioblastoma U251 cells. Oncol. Lett..

[12] Kalimutho M., Sinha D., Jeffery J., Nones K., Srihari S., Fernando W.C., Duijf P.H., Vennin C., Raninga P., Nanayakkara D. (2018). CEP 55 is a determinant of cell fate during perturbed mitosis in breast cancer. EMBO Mol. Med..

[13] Tang Z., Kang B., Li C., Chen T., Zhang Z. (2019). GEPIA2: An enhanced web server for large-scale expression profiling and interactive analysis. Nucleic Acids Res..

[14] Pontén F., Jirström K., Uhlen M. (2008). The Human Protein Atlas--a tool for pathology. J. Pathol..

[15] Ru B., Wong C.N., Tong Y., Zhong J.Y., Zhong S.S.W., Wu W.C., Chu K.C., Wong C.Y., Lau C.Y., Chen I. (2019). TISIDB: An integrated repository portal for tumor–immune system interactions. Bioinformatics.

[16] Bartha Á., Győrffy B. (2021). TNMplot.com: A Web Tool for the Comparison of Gene Expression in Normal, Tumor and Metastatic Tissues. Int. J. Mol. Sci..

[17] Chandrashekar D.S., Karthikeyan S.K., Korla P.K., Patel H., Shovon A.R., Athar M., Netto G.J., Qin Z.S., Kumar S., Manne U. (2022). UALCAN: An update to the integrated cancer data analysis platform. Neoplasia.

[18] Pontén F., Schwenk J.M., Asplund A., Edqvist P.-H.D. (2011). The Human Protein Atlas as a proteomic resource for biomarker discovery. J. Intern. Med..

[19] Gao J., Aksoy B.A., Dogrusoz U., Dresdner G., Gross B.E., Sumer S.O., Sun Y., Jacobsen A., Sinha R., Larsson E. (2013). Integrative Analysis of Complex Cancer Genomics and Clinical Profiles Using the cBioPortal. Sci. Signal..

[20] Yang X., Han H., De Carvalho D.D., Lay F.D., Jones P.A., Liang G. (2014). Gene Body Methylation Can Alter Gene Expression and Is a Therapeutic Target in Cancer. Cancer Cell.

[21] Cai Z., Li C.-F., Han F., Liu C., Zhang A., Hsu C.-C., Peng D., Zhang X., Jin G., Rezaeian A.-H. (2020). Phosphorylation of PDHA by AMPK Drives TCA Cycle to Promote Cancer Metastasis. Mol. Cell.

[22] Li Y., Ge D., Lu C. (2019). The SMART App: An interactive web application for comprehensive DNA methylation analysis and visualization. Epigenetics Chromatin.

[23] Yang Y., Li C., Liu T., Dai X., Bazhin A.V. (2020). Myeloid-Derived Suppressor Cells in Tumors: From Mechanisms to Antigen Specificity and Microenvironmental Regulation. Front. Immunol..

[24] Liu T., Han C., Wang S., Fang P., Ma Z., Xu L., Yin R. (2019). Cancer-associated fibroblasts: An emerging target of anti-cancer immunotherapy. J. Hematol. Oncol..

[25] Li T., Fu J., Zeng Z., Cohen D., Li J., Chen Q., Li B., Liu X.S. (2020). TIMER2.0 for analysis of tumor-infiltrating immune cells. Nucleic Acids Res..

[26] Hargadon K.M., Johnson C.E., Williams C.J. (2018). Immune checkpoint blockade therapy for cancer: An overview of FDA-approved immune checkpoint inhibitors. Int. Immunopharmacol..

[27] Li N., Liang X., Li J., Zhang D., Li T., Guo Z. (2021). C-C motif chemokine ligand 14 inhibited colon cancer cell proliferation and invasion through suppressing M2 polarization of tumor-associated macrophages. Histol. Histopathol..

[28] Nie G., Cao X., Mao Y., Lv Z., Lv M., Wang Y., Wang H., Liu C. (2021). Tumor-associated macrophages-mediated CXCL8 infiltration enhances breast cancer metastasis: Suppression by Danirixin. Int. Immunopharmacol..

[29] Rizzo A., Ricci A.D., Brandi G. (2021). PD-L1, TMB, MSI, and Other Predictors of Response toImmune Checkpoint Inhibitors in Biliary Tract Cancer. Cancers.

[30] Shen W., Song Z., Zhong X., Huang M., Shen D., Gao P., Qian X., Wang M., He X., Song X. (2022). Sangerbox: A comprehensive, interaction-friendly clinical bioinformatics analysis platform. iMeta.

[31] Abotaleb M., Kubatka P., Caprnda M., Varghese E., Zolakova B., Zubor P., Opatrilova R., Kruzliak P., Stefanicka P., Büsselberg D. (2018). Chemotherapeutic agents for the treatment of metastatic breast cancer: An update. Biomed. Pharmacother..

[32] Reinhold W.C., Sunshine M., Liu H., Varma S., Kohn K.W., Morris J., Doroshow J., Pommier Y. (2012). CellMiner: A web-based suite of genomic and pharmacologic tools to explore transcript and drug patterns in the NCI-60 cell line set. Cancer Res..

[33] Szklarczyk D., Gable A.L., Nastou K.C., Lyon D., Kirsch R., Pyysalo S., Doncheva N.T., Legeay M., Fang T., von Mering C. (2021). The STRING database in 2021: Customizable protein-protein networks, and functional characterization of user-uploaded gene/measurement sets. Nucleic Acids Res..

[34] Sherman B.T., Hao M., Qiu J., Jiao X., Baseler M.W., Lane H.C., Imamichi T., Chang W. (2022). DAVID: A web server for functional enrichment analysis and functional annotation of gene lists (2021 update). Nucleic Acids Res..

[35] Peart O. (2015). Breast intervention and breast cancer treatment options. Radiol Technol..

[36] Fabbro M., Zhou B.B., Takahashi M., Sarcevic B., Lal P., Graham M.E., Gabrielli B.G., Robinson P.J., Nigg E.A., Khanna K.K. (2005). Cdk1/Erk2- and Plk1-dependent phosphorylation of a centrosome protein, Cep55, is required for its recruitment to midbody and cytokinesis. Dev Cell..

[37] Tao J., Zhi X., Tian Y., Li Z., Zhu Y., Wang W., Xie K., Tang J., Zhang X., Wang L. (2014). CEP55 contributes to human gastric carcinoma by regulating cell proliferation. Tumor Biol..

[38] Yang Y.-F., Zhang M.-F., Tian Q.-H., Fu J., Yang X., Zhang C.Z., Yang H. (2018). SPAG5 interacts with CEP55 and exerts oncogenic activities via PI3K/AKT pathway in hepatocellular carcinoma. Mol. Cancer.

[39] Li F., Jin D., Tang C., Gao D. (2018). CEP55 promotes cell proliferation and inhibits apoptosis via the PI3K/Akt/p21 signaling pathway in human glioma U251 cells. Oncol. Lett..

[40] Xu L., Xia C., Sheng F., Sun Q., Xiong J., Wang S. (2018). CEP55 promotes the proliferation and invasion of tumour cells via the AKT signalling pathway in osteosarcoma. Carcinogenesis.

[41] Jia Y., Xiao Z., Gongsun X., Xin Z., Shang B., Chen G., Wang Z., Jiang W. (2018). CEP55 promotes the proliferation, migration and invasion of esophageal squamous cell carcinoma via the PI3K/Akt pathway. OncoTargets Ther..

[42] Li M., Gao J., Li D., Yin Y. (2018). CEP55 promotes cell motility via JAK2–STAT3– MMPs cascade in hepatocellular carcinoma. Cells.

[43] Yang L., He Y., Zhang Z., Wang W. (2020). Upregulation of CEP55 Predicts Dismal Prognosis in Patients with Liver Cancer. BioMed Res. Int..

[44] Jiang C., Zhang Y., Li Y., Lu J., Huang Q., Xu R., Feng Y., Yan S. (2018). High CEP55 expression is associated with poor prognosis in non-small-cell lung cancer. OncoTargets Ther..

[45] Jiang W., Wang Z., Jia Y. (2017). CEP55 overexpression predicts poor prognosis in patients with locally advanced esophageal squamous cell carcinoma. Oncol. Lett..

[46] Zhang W., Niu C., He W., Hou T., Sun X., Xu L., Zhang Y. (2016). Upregulation of centrosomal protein 55 is associated with unfavorable prognosis and tumor invasion in epithelial ovarian carcinoma. Tumor Biol..

[47] Wang Y., Jin T., Dai X., Xu J. (2016). Lentivirus-mediated knockdown of CEP55 suppresses cell proliferation of breast cancer cells. Biosci. Trends.

[48] Li M., Liu Y., Jiang X., Hang Y., Wang H., Liu H., Chen Z., Xiao Y. (2021). Inhibition of miR-144-3p exacerbates non-small cell lung cancer progression by targeting CEP55. Acta Biochim. Biophys. Sin..

[49] Bayarkhangai B., Noureldin S., Yu L., Zhao N., Gu Y., Xu H., Guo C. (2018). A comprehensive and perspective view of oncoprotein SET in cancer. Cancer Med..

[50] Telloni S.M. (2017). Tumor Staging and Grading: A Primer. Methods Mol. Biol..

[51] Fares J., Fares M.Y., Khachfe H.H., Salhab H.A., Fares Y. (2020). Molecular principles of metastasis: A hallmark of cancer revisited. Signal Transduct. Target. Ther..

[52] Nagy Á., Munkácsy G., Győrffy B. (2021). Pancancer survival analysis of cancer hallmark genes. Sci. Rep..

[53] Zhang X., Xu Q., Li E., Shi T., Chen H. (2022). CEP55 predicts the poor prognosis and promotes tumorigenesis in endometrial cancer by regulating the Foxo1 signaling. Mol. Cell. Biochem..

[54] Fu Y., Dominissini D., Rechavi G., He C. (2014). Gene expression regulation mediated through reversible m6A RNA methylation. Nat. Rev. Genet..

[55] Feng L.Y., Chen C.X., Li L. (2019). Hypermethylation of tumor suppressor genes is a risk factor for poor prognosis in ovarian cancer A meta-analysis. Medicine.

[56] Chen Y., Wang D., Peng H., Chen X., Han X., Yu J., Wang W., Liang L., Liu Z., Zheng Y. (2019). Epigenetically upregulated oncoprotein PLCE1 drives esophageal carcinoma angiogenesis and proliferation via activating the PI-PLCϵ-NF-κB signaling pathway and VEGF-C/ Bcl-2 expression. Mol. Cancer.

[57] Anglim P.P., A Alonzo T., A Laird-Offringa I. (2008). DNA methylation-based biomarkers for early detection of non-small cell lung cancer: An update. Mol. Cancer.

[58] Shao C., Sun W., Tan M., Glazer C.A., Bhan S., Zhong X., Fakhry C., Sharma R., Westra W.H., Hoque M.O. (2011). Integrated, Genome-Wide Screening for Hypomethylated Oncogenes in Salivary Gland Adenoid Cystic Carcinoma. Clin. Cancer Res..

[59] Hur K., Cejas P., Feliu J., Moreno-Rubio J., Burgos E., Boland C.R., Goel A. (2014). Hypomethylation of long interspersed nuclear element-1 (LINE-1) leads to activation of proto-oncogenes in human colorectal cancer metastasis. Gut.

[60] Søes S., Daugaard I.L., Sørensen B.S., Carus A., Mattheisen M., Alsner J., Overgaard J., Hager H., Hansen L.L., Kristensen L.S. (2014). Hypomethylation and increased expression of the putative oncogene ELMO3 are associated with lung cancer development and metastases formation. Oncoscience.

[61] Ardito F., Giuliani M., Perrone D., Troiano G., Muzio L.L. (2017). The crucial role of protein phosphorylation in cell signalingand its use as targeted therapy (Review). Int. J. Mol. Med..

[62] Yang X., Zhong W., Cao R. (2020). Phosphorylation of the mRNA cap-binding protein eIF4E and cancer. Cell. Signal..

[63] Babu N., Pinto S.M., Biswas M., Subbannayya T., Rajappa M., Mohan S.V., Advani J., Rajagopalan P., Sathe G., Syed N. (2020). Phosphoproteomic analysis identifies CLK1 as a novel therapeutic target in gastric cancer. Gastric Cancer.

[64] Ponath V., Frech M., Bittermann M., Al Khayer R., Neubauer A., Brendel C., Von Strandmann E.P. (2020). The Oncoprotein SKI Acts as A Suppressor of NK Cell-Mediated Immunosurveillance in PDAC. Cancers.

[65] Gunaydin G., Kesikli S.A., Guc D. (2015). Cancer associated fibroblasts have phenotypic and functional characteristics similar to the fibrocytes that represent a novel MDSC subset. Oncoimmunology.

[66] Umansky V., Blattner C., Gebhardt C., Utikal J. (2016). The Role of Myeloid-Derived Suppressor Cells (MDSC) in Cancer Progression. Vaccines.

[67] Deng X., Li X., Guo X., Lu Y., Xie Y., Huang X., Lin J., Tan W., Wang C. (2022). Myeloid-derived suppressor cells promote tumor growth and sorafenib resistance by inducing FGF1 upregulation and fibrosis. Neoplasia.

[68] Attieh Y., Vignjevic D.M. (2016). The hallmarks of CAFs in cancer invasion. Eur. J. Cell Biol..

[69] Kobayashi H., Enomoto A., Woods S.L., Burt A.D., Takahashi M., Worthley D.L. (2019). Cancer-associated fibroblasts in gastrointestinal cancer. Nat. Rev. Gastroenterol. Hepatol..

[70] Jiang W., He Y., He W., Wu G., Zhou X., Sheng Q., Zhong W., Lu Y., Ding Y., Lu Q. (2021). Exhausted CD8+T Cells in the Tumor Immune Microenvironment: New Pathways to Therapy. Front. Immunol..

[71] Zeng E.A.Z., Wei F., Ren X. (2020). Exhausted T cells and epigenetic status. Cancer Biol. Med..

[72] de Moura R.G., Covre L.P., Fantecelle C.H., Gajardo V.A.T., Cunha C.B., Stringari L.L., Belew A.T., Daniel C.B., Von Zeidler S.V., Gomes D.C.O. (2021). PD-1 Blockade Modulates Functional Activities of Exhausted-Like T Cell in Patients With Cutaneous Leishmaniasis. Front. Immunol..

[73] Fang W., Ye L., Shen L., Cai J., Huang F., Wei Q., Fei X., Chen X., Guan H., Wang W. (2014). Tumor-associated macrophages promote the metastatic potential of thyroid papillary cancer by releasing CXCL8. Carcinogenesis.

[74] Hosono M., Koma Y.-I., Takase N., Urakawa N., Higashino N., Suemune K., Kodaira H., Nishio M., Shigeoka M., Kakeji Y. (2017). CXCL8 derived from tumor-associated macrophages and esophageal squamous cell carcinomas contributes to tumor progression by promoting migration and invasion of cancer cells. Oncotarget.

[75] Zhu M., Xu W., Wei C., Huang J., Xu J., Zhang Y., Zhao Y., Chen J., Dong S., Liu B. (2019). CCL14 serves as a novel prognostic factor and tumor suppressor of HCC by modulating cell cycle and promoting apoptosis. Cell Death Dis..

[76] Cai Y., Ling Y., Huang L., Huang H., Chen X., Xiao Y., Zhu Z., Chen J. (2020). C-C motif chemokine 14 as a novel potential biomarker for predicting the prognosis of epithelial ovarian cancer. Oncol. Lett..

[77] Yang Z., Li C., Yan C., Li J., Yan M., Liu B., Zhu Z., Wu Y., Gu Q. (2019). KIF14 promotes tumor progression and metastasis and is an independent predictor of poor prognosis in human gastric cancer. Biochim. Biophys. Acta BBA Mol. Basis Dis..

[78] Singel S.M., Cornelius C., Zaganjor E., Batten K., Sarode V.R., Buckley D.L., Peng Y., John G.B., Li H.C., Sadeghi N. (2014). KIF14 Promotes AKT Phosphorylation and Contributes to Chemoresistance in Triple-Negative Breast Cancer. Neoplasia.

[79] Zhang Y., Yuan Y., Liang P., Zhang Z., Guo X., Xia L., Zhao Y., Shu X.-S., Sun S., Ying Y. (2017). Overexpression of a novel candidate oncogene KIF14 correlates with tumor progression and poor prognosis in prostate cancer. Oncotarget.

[80] Li X.-L., Ji Y.-M., Song R., Li X.-N., Guo L.-S. (2019). KIF23 Promotes Gastric Cancer by Stimulating Cell Proliferation. Dis. Markers.

[81] Liu Y., Chen H., Dong P., Xie G., Zhou Y., Ma Y., Yuan X., Yang J., Han L., Chen L. (2020). KIF23 activated Wnt/β-catenin signaling pathway through direct interaction with Amer1 in gastric cancer. Aging.

[82] Jian W., Deng X.-C., Munankarmy A., Borkhuu O., Ji C.-L., Wang X.-H., Zheng W.-F., Yu Y.-H., Zhou X.-Q., Fang L. (2021). KIF23 promotes triple negative breast cancer through activating epithelial-mesenchymal transition. Gland. Surg..

[83] Chen J., Xia H., Zhang X., Karthik S., Pratap S.V., Ooi L.L., Hong W., Hui K.M. (2015). ECT2 regulates the Rho/ERK signalling axis to promote early recurrence in human hepatocellular carcinoma. J. Hepatol..

[84] Jin Y., Yu Y., Shao Q., Ma Y., Zhang R., Yao H., Xu Y. (2014). Up-regulation of ECT2 is associated with poor prognosis in gastric cancer patients. Int. J. Clin. Exp. Pathol..

